# Emergency Department Prediction of In-Hospital Mortality in Suspected Pulmonary Embolism: An Explainable Machine Learning Approach

**DOI:** 10.3390/jcm15041340

**Published:** 2026-02-08

**Authors:** Meliha Fındık, Tufan Alatlı, Salih Kocaoğlu, Yeltuğ Esra Gelen, Rahime Sema Taş

**Affiliations:** 1 Department of Emergency Medicine, Balikesir University, 10145 Balikesir, Türkiye; drtufanalatli@gmail.com (T.A.); salihkocaoglu1986@gmail.com (S.K.); rsematas@gmail.com (R.S.T.); 2 Sinop Atatürk State Hospital, 57000 Sinop, Türkiye; bilenessra@gmail.com

**Keywords:** pulmonary embolism, emergency department, machine learning, mortality prediction, explainable AI, SHAP, risk stratification

## Abstract

**Background:** Pulmonary embolism (PE) is a significant cause of cardiovascular mortality, and emergency department (ED) management requires early risk assessment to guide monitoring and disposition. Because key decisions are often needed while diagnostic evaluation is ongoing, the simplified Pulmonary Embolism Severity Index (sPESI) may provide limited discrimination for in-hospital outcomes. We evaluated whether explainable machine-learning (ML) models integrating routine ED variables with validated risk scores can predict in-hospital mortality in adults evaluated for suspected acute PE. **Methods:** A retrospective single-center cohort study was performed, including 220 consecutive adults evaluated for suspected acute PE in the ED between January 2021 and March 2025, comprising both PE-confirmed and PE-excluded cases. Predictors included demographics, vital signs, arterial blood gas indices, available imaging/echocardiographic findings, and Wells, Revised Geneva, and sPESI scores. Seven ML algorithms were trained and internally evaluated using the area under the receiver operating characteristic curve (AUC) and complementary metrics. Model interpretability was assessed using SHAP (SHAPley Additive exPlanations), and a sensitivity analysis was conducted in the PE-confirmed subgroup. **Results:** Tree-based ensemble models demonstrated higher discrimination for in-hospital all-cause mortality than simpler classifiers. SHAP analyses consistently highlighted sPESI, oxygenation/arterial blood gas indices, and malignancy as key contributors to mortality risk. Findings were similar in the PE-confirmed sensitivity analysis. **Conclusions:** Explainable ML models combining established risk scores with routinely collected ED variables may complement risk stratification along the suspected-PE pathway. External multicenter validation and prospective impact studies are warranted before clinical implementation.

## 1. Introduction

Pulmonary embolism (PE) is the third most common cause of mortality among cardiovascular diseases and accounts for a substantial proportion of in-hospital deaths worldwide [1]. Its epidemiology, natural history, and risk factors have long been recognized, with classical reviews characterizing PE as both common and potentially fatal, with persistently high early mortality rates [2,3]. The clinical presentation of acute PE is highly heterogeneous, ranging from asymptomatic or mild disease to life-threatening hemodynamic instability [4]. Diagnostic delays are common owing to nonspecific symptoms, and such delays are associated with increased morbidity and mortality [5].

To support clinical decision-making in suspected PE, several clinical probability scores have been developed, including the Wells, Geneva, and Revised Geneva models. Although these tools are useful for estimating pre-test probability and guiding diagnostic work-up, their primary purpose is diagnostic rather than prognostic, and they include subjective components that may not fully reflect acute disease severity [6,7]. For prognostic purposes, the Pulmonary Embolism Severity Index (PESI) and simplified PESI (sPESI) are widely used to predict 30-day mortality based on vital signs and comorbidities [8]. However, intermediate-risk patients remain challenging to stratify because conventional scores are primarily based on baseline clinical variables and comorbidities and do not routinely integrate imaging findings, cardiac biomarkers, or dynamic physiological changes relevant to early decompensation [4,9]. Recent advances, including updated risk stratification models, Pulmonary Embolism Response Teams (PERTs), and catheter-directed interventions, have expanded therapeutic options; nevertheless, early identification of patients at risk of hemodynamic decompensation remains difficult [10].

Computed tomography pulmonary angiography (CTPA) is considered the gold standard for diagnosing PE because it provides direct visualization of thromboembolic obstruction [11]. However, its routine use may be limited by contraindications such as renal dysfunction, concerns regarding contrast-associated kidney injury, and technical artifacts that can reduce image quality and diagnostic confidence [12]. Moreover, advanced age, underlying malignancies, and chronic lung disease may further reduce diagnostic certainty and complicate image interpretation. Ventilation/perfusion (V/Q) imaging, including PET/CT, retains diagnostic value in selected patients—particularly when CTPA findings are nondiagnostic or equivocal—and has been shown to improve confidence in such cases [13]. Therefore, although CTPA remains the primary diagnostic modality, its practical limitations underscore the need for complementary strategies to support early risk assessment when definitive imaging is delayed or inconclusive.

In recent years, machine learning (ML) algorithms have shown promise in predicting both PE diagnosis and clinical outcomes by analyzing multidimensional clinical, laboratory, and imaging data. By capturing complex nonlinear relationships and high-order interactions, ML models may identify prognostic patterns that traditional clinical scores can overlook, and several studies have reported improved predictive performance with ML-based approaches [14,15]. Furthermore, explainable artificial intelligence (XAI) techniques such as SHAP increase the interpretability of model outputs, which is essential for clinical adoption [1,16].

Nevertheless, the field remains controversial. While some investigators argue that robust ML performance requires large, multicenter datasets enriched with advanced biomarkers or comprehensive imaging inputs, others suggest that combining routinely available emergency department (ED) data with established clinical scores can yield accurate, clinically interpretable models for early risk assessment. Recent studies employing deep learning and multimodal approaches have demonstrated improved classification and prognostic performance compared with traditional methods [5,17]. In parallel, ML models developed using only data available at ED presentation—without relying on immediate CTPA—have shown potential utility for early decision support in suspected PE, broadening applicability in acute care settings.

In recent years, there has also been growing interest in using ML approaches for PE across various domains. Several studies have applied ML models to large clinical datasets for mortality prediction, reporting improved performance over conventional clinical scores while incorporating modern explainability techniques [18]. Deep learning models have demonstrated high accuracy for automated PE detection on CTPA, although these systems rely predominantly on imaging data and often lack integration with clinical variables relevant to bedside decision-making [19,20,21]. Other investigations have evaluated ED-based ML tools to assist diagnostic assessment before imaging; however, these models have not examined short-term outcomes, such as mortality [22]. Despite these developments, systematic reviews continue to identify substantial limitations—including inconsistent reporting standards, limited dataset transparency, and insufficient external validation—which restrict the clinical implementation of existing AI tools [23,24,25].

Artificial intelligence is increasingly embedded in clinical decision support systems, yet limited transparency, trust, and workflow remain major barriers to real-world uptake [26]. Evidence syntheses of explainable AI highlight that post hoc tools such as SHAP can improve interpretability but may introduce usability challenges, including explanation consistency, cognitive load, and mismatch with clinicians’ mental models [26,27].

Accordingly, there remains a need for interpretable ML models that can deliver reliable early mortality risk estimates using variables routinely accessible during ED evaluation of suspected PE. This study aimed to compare multiple ML algorithms for predicting in-hospital all-cause mortality in adults evaluated for suspected acute PE, to identify the best-performing model, and to establish an explainable framework suitable for clinical decision support.

## 2. Materials and Methods

### 2.1. Study Design and Data Collection

This retrospective cohort study was conducted at the Department of Emergency Medicine, Balıkesir University Faculty of Medicine, between 1 January 2021, and 30 March 2025. Data were extracted from the hospital’s electronic medical record system and archived patient records. The study population consisted of consecutive adults evaluated for suspected PE who underwent computed tomography pulmonary angiography (CTPA).

**Figure 1:**
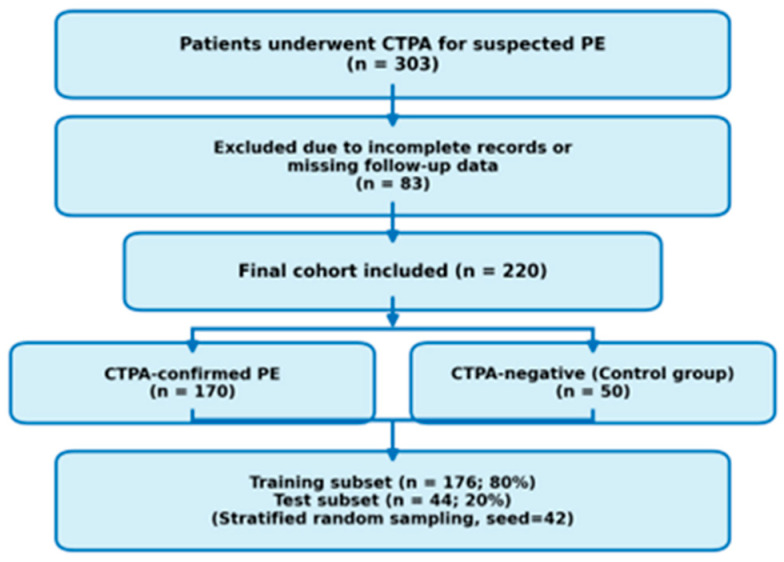
Patient selection and dataset allocation.

During the study period, 303 adults who underwent CTPA for suspected PE were screened. Exclusions were due to incomplete clinical/imaging data (*n* = 83) and/or to in-hospital mortality follow-up being unavailable; most reflected non-retrievable predictors/outcomes related to inter-facility transfers and fragmented documentation. D-dimer, troponin, and NT-proBNP were not included because they were not ordered systematically (substantial non-random missingness); including them would have reduced the analytic sample and introduced selective-testing bias. The final cohort comprised 220 patients: 170 CTPA-confirmed PE and 50 patients in whom PE was excluded. Primary analyses were conducted in the overall suspected-PE cohort (*n* = 220), with a prespecified sensitivity analysis in confirmed PE (*n* = 170). PE-excluded patients were retained to reflect the ED “suspected PE” pathway and were reported separately to avoid conflating diagnostic and prognostic inference (Figure 1).

Data verification was performed independently by two investigators. Collected variables included demographics (age, sex); medical history (prior PE or venous thromboembolism [VTE], malignancy, recent surgery, immobilization); clinical findings (systolic and diastolic blood pressure, heart rate, hemoptysis); laboratory data (arterial blood gas parameters: pO_2_, pCO_2_); and cardiac imaging results (electrocardiography [ECG] and echocardiography [ECHO]). When available, imaging findings were abstracted from contemporaneous ED documentation and reports.

Additionally, the following validated clinical scores were retrospectively calculated: the Wells score, the Revised Geneva score, and the Simplified Pulmonary Embolism Severity Index (sPESI). These scores were included as candidate predictors and were also used as reference comparators in benchmarking analyses. These scores were not systematically recorded in the routine ED workflow, and when present, they were inconsistently documented. Therefore, we recalculated all scores for all patients from structured fields and contemporaneous ED notes using prespecified rules. For subjective Wells items, we relied only on explicit documentation; otherwise, the item was coded conservatively as absent. All parameters were recorded at ED presentation before anticoagulation initiation or transfer to an inpatient unit.

### 2.2. Machine Learning Framework

Feature preprocessing and model development were performed in Python (v3.11.x; Python Software Foundation, Beaverton, OR, USA) using scikit-learn (vX.Y.Z; scikit-learn developers; Inria, Paris, France), XGBoost (vX.Y.Z; XGBoost Contributors; DMLC/University of Washington, Seattle, WA, USA), LightGBM (vX.Y.Z; Microsoft Corporation, One Microsoft Way, Redmond, WA, USA), and CatBoost (vX.Y.Z; Yandex LLC, Ulitsa Lva Tolstogo 16, Moscow, Russia). To minimize information leakage, preprocessing steps were applied only to the training data and then used to transform the validation and test sets. During five-fold cross-validation, the same preprocessing pipeline was refitted within each training fold and then applied to the corresponding validation fold. All numerical variables were standardized using z-score normalization, and categorical features were encoded as binary variables. Missing data (<5%) were imputed using median values, and cases lacking mortality information were excluded.

The dataset was split into training (80%) and test (20%) subsets using stratified sampling to preserve the distribution of the target outcome (in-hospital mortality). A fixed random seed (random_state = 42) was used to ensure reproducibility. The training set was used for model development and hyperparameter optimization, whereas the held-out test set was reserved for final performance evaluation.

Seven supervised machine-learning algorithms were trained and compared: Extreme Gradient Boosting (XGBoost), Random Forest (RF), CatBoost, Light Gradient Boosting Machine (LightGBM), Logistic Regression (LR), Support Vector Machine (SVM) with a radial basis function (RBF) kernel, and K-Nearest Neighbors (KNN). Hyperparameter tuning was performed using a 5-fold cross-validation grid search on the training set, with AUC as the primary optimization metric. Cross-validation results were used for model selection and tuning, and the test set was reserved for final evaluation.

Feature selection and dimensionality reduction: No separate feature selection or dimensionality reduction procedure was applied prior to model training. All prespecified candidate predictors were retained, and model-embedded mechanisms (e.g., regularization and tree-based importance) were used, along with SHAP, to quantify each feature’s relative contribution.

Class imbalance handling: Because survival occurred more frequently than mortality, class-weighted learning was applied where supported (e.g., class_weight for LR/SVM and equivalent weighting strategies for tree-based models). In addition to ROC-AUC, precision–recall analysis and threshold-dependent metrics (precision, recall, and F1-score) were reported to provide a complementary assessment under unequal class frequencies.

#### 2.2.1. Workflow Overview

**Figure 2:**
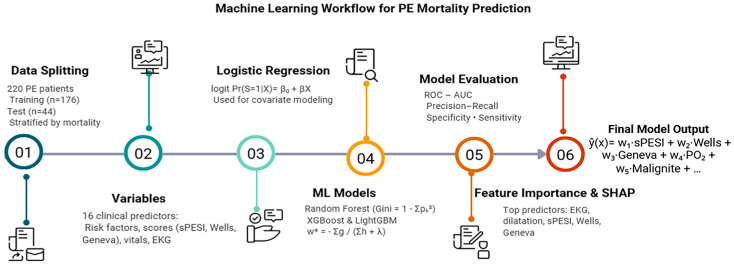
Machine Learning Workflow for PE Mortality Prediction.

The overall ML workflow, including data preparation, model training, hyperparameter optimization, and SHAP-based interpretability, is presented in the flowchart provided in Figure 2. The workflow outlines each procedural step, from initial data preprocessing to final model evaluation, to ensure methodological transparency and reproducibility. Briefly, the pipeline comprised (i) data cleaning and missingness handling, (ii) leakage-free preprocessing (fit on training data/within CV folds; apply to validation/test), (iii) stratified train–test split (80/20; random_state = 42), (iv) model training and hyperparameter optimization via five-fold cross-validation on the training set, (v) final evaluation on the held-out test set, and (vi) SHAP-based interpretability analyses.

#### 2.2.2. Mathematical Formulation of ML Models

To increase methodological transparency, the mathematical definitions of the ML algorithms used are given below.

Dataset and Notation: For each patient *i* in the dataset (*n* = 220), the outcome variable Yᵢ ∈ {0,1} was defined as follows: *Y*ᵢ = 1 indicates in-hospital mortality, and *Y*ᵢ = 0 indicates survival. The predictor vector was denoted as *X*ᵢ = (*x*ᵢ_1_, …, *x*ᵢₚ), where *p* denotes the number of input features included in model development. The dataset was stratified-sampled into a training set (*n* = 176) and a held-out test set (*n* = 44).

Logistic Regression (LR):

$$\mathrm{logit}(\pi_{i})=log(\frac{\pi_{i}}{1-\pi_{i}})=\beta_{0}+\sum_{j=1}^{16}\beta_{j}x_{ij}$$

$${\hat{\pi }}_{i}=\frac{1}{1+exp(-\beta_{0}-\sum \beta_{j}x_{ij})}$$

Random Forest (RF)*:* A random forest consists of *T* decision trees, each trained on a bootstrap sample. The predicted mortality probability is the average over all trees:

$${\hat{\pi }}_{i}^{RF}=\frac{1}{T}\sum_{t=1}^{T}f_{t}(X_{i})$$

Boosting Models (XGBoost/LightGBM): Gradient boosting constructs an additive model of *K* weak learners:

$$\hat{F}(X_{i})=\sum_{k=1}^{K}f_{k}(X_{i})$$

and the probability estimate is:

$${\hat{\pi }}_{i}=\frac{1}{1+e^{-\hat{F}(X_{i})}}$$

Each boosting step fits a new function faltk by minimizing the loss function:

fk=argminf∑il(Yi, F^k−1(Xi)+f(Xi))

Classification Threshold: A predicted probability is converted into a binary classification using:

$${\hat{Y}}_{i}=\{\begin{array}{cc} 1, & {\hat{\pi }}_{i}\geq c\\ 0, & {\hat{\pi }}_{i}<c \end{array}$$

Performance Metrics

$$\mathrm{Accuracy}=\frac{TP+TN}{TP+TN+FP+FN}$$

$$\mathrm{Sensitivity}=\frac{TP}{TP+FN}$$

$$\mathrm{Specificity}=\frac{TN}{TN+FP}$$

$$\mathrm{Brier}\;\mathrm{Score}=\frac{1}{n_{\mathrm{test}}}\sum_{i}(Y_{i}-{\hat{\pi }}_{i}{)}^{2}$$

### 2.3. Model Evaluation and Explainability

Model performance was evaluated using accuracy, area under the receiver operating characteristic curve (AUC), recall (sensitivity), specificity, precision (positive predictive value), F1-score, and negative predictive value. To provide a robust assessment under class imbalance, precision–recall curves and corresponding metrics were also reported. Additional evaluation included confusion matrices and calibration performance. Threshold-dependent metrics were computed using a 0.50 probability threshold to enable standardized comparisons across models. However, the operating threshold can be adjusted according to clinical priorities, and discrimination was primarily assessed using threshold-agnostic metrics, including ROC-AUC and PR-AUC; ROC and precision–recall curves were therefore used to illustrate performance across a range of thresholds.

For interpretability, SHapley Additive exPlanations (SHAP) analysis was applied to quantify each variable’s contribution to mortality prediction. Feature importance rankings were derived from SHAP summary values combined with internal model coefficients, providing both global and local interpretability.

Five-fold cross-validation on the training set was used for hyperparameter optimization and to summarize variability in training-phase performance. In contrast, final model performance was reported on the held-out test set. Model performance was further illustrated by receiver operating characteristic and precision–recall curves, confusion matrices, calibration plots, and learning curves.

Decision curve analysis was performed to evaluate clinical utility by estimating net benefit across threshold probabilities and comparing the ML model with treat-all, treat-none, and a score-only sPESI model. Calibration was assessed using calibration plots of observed versus predicted probabilities and Brier scores, using five-fold out-of-fold predicted probabilities.

### 2.4. Statistical Analysis

Continuous variables were summarized as mean ± standard deviation (SD) or median (interquartile range [IQR]), and categorical variables as frequencies (percentages). Normality of continuous variables was assessed using the Shapiro–Wilk test. Between-group comparisons (survivors vs. non-survivors) were performed using the independent-samples t-test or Mann–Whitney U test for continuous variables, and the Chi-square test or Fisher’s exact test for categorical variables, as appropriate. A *p*-value < 0.05 was considered statistically significant. All statistical analyses were performed in Python (v3.11) using pandas and scipy.

AI-assisted language editing was used to improve grammar, clarity, and readability; all scientific content, analyses, and final wording were reviewed and approved by the authors.

## 3. Results

### 3.1. Patient and Data Characteristics

A total of 220 patients were included after data validation. Of these, 176 were allocated to the training set and 44 to the test set using a stratified train–test split. The overall selection procedure and dataset distribution are shown in Figure 1. Baseline demographic, clinical, and laboratory characteristics of the training and test subsets are presented in Table 1.

There were no statistically significant differences between the training and test subsets across demographic, clinical, or laboratory variables (*p* > 0.05 for all comparisons). Age, sex distribution, comorbidities, vital signs, arterial blood gas parameters, echocardiographic findings, and clinical risk scores were comparable between subsets. The stratified split maintained a similar prevalence of in-hospital mortality across both sets, supporting the model’s internal consistency during development and evaluation.

### 3.2. Model Performance

**Figure 3:**
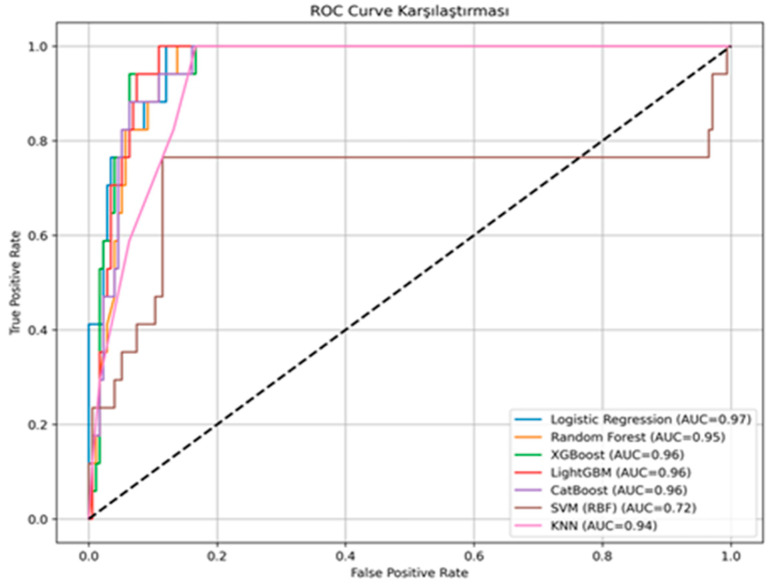
ROC curves for in-hospital mortality prediction in adults evaluated for suspected acute pulmonary embolism using seven machine learning algorithms.

Seven machine learning algorithms were evaluated to predict in-hospital mortality. ROC curves are shown in Figure 3. Ensemble-based classifiers, including Random Forest, CatBoost, XGBoost, and LightGBM, demonstrated the highest discriminative performance, with AUC values ranging from 0.870 to 0.880. The k-nearest neighbors model showed moderate discrimination, with an AUC of 0.850. By contrast, the support vector machine with a radial basis function kernel demonstrated limited discriminative power, with an AUC of 0.530.

**Figure 4:**
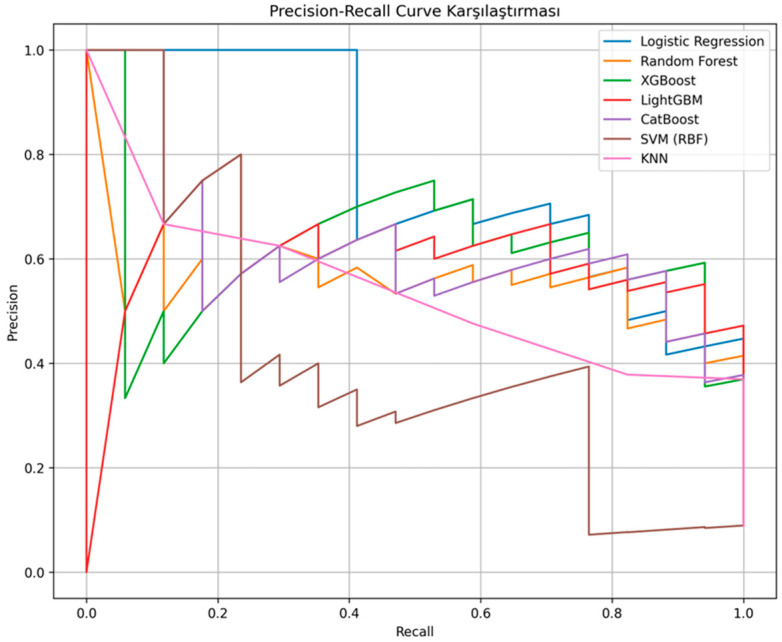
Precision–recall curves for in-hospital mortality prediction in adults evaluated for suspected acute pulmonary embolism.

**Figure 5:**
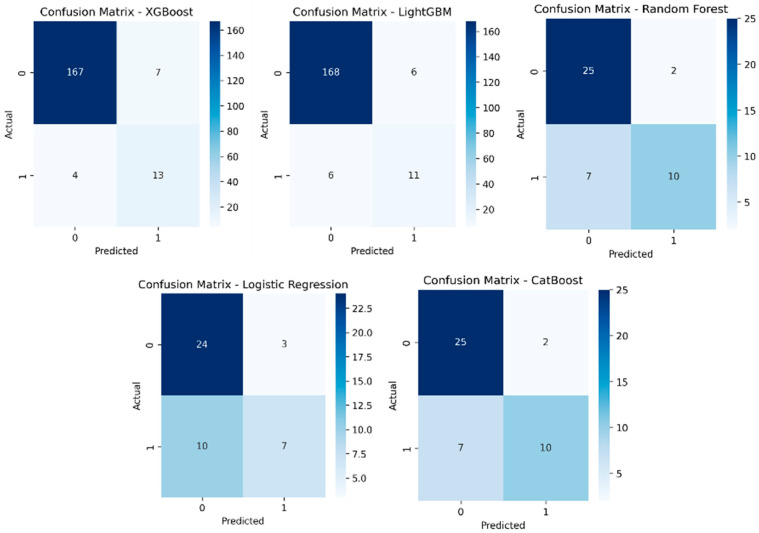
Confusion matrices of selected machine learning models, illustrating accuracy, specificity, precision, and recall performance.

Precision–recall curves are shown in Figure 4, and confusion matrices are presented in Figure 5. Random Forest achieved the highest accuracy of 79.5%, with specificity of 92.6%, precision of 83.3%, recall of 58.8%, and an F1-score of 69.0%. CatBoost demonstrated the same overall performance, with accuracy of 79.5%, specificity of 92.6%, precision of 83.3%, recall of 58.8%, and an F1-score of 69.0%. XGBoost and LightGBM both achieved 77.3% accuracy and an AUC of 0.880. Logistic Regression attained an accuracy of 70.5%, an AUC of 0.880, and a recall of 41.2%. The k-nearest neighbors model achieved an accuracy of 65.9%, an AUC of 0.850, and a recall of 41.25%. The support vector machine model with a radial basis function kernel achieved an accuracy of 61.4%, an AUC of 0.530, a specificity of 100%, and a recall of 0%. A detailed comparison of performance metrics is provided in Table 2.

### 3.3. Feature Importance and Explainability

**Figure 6:**
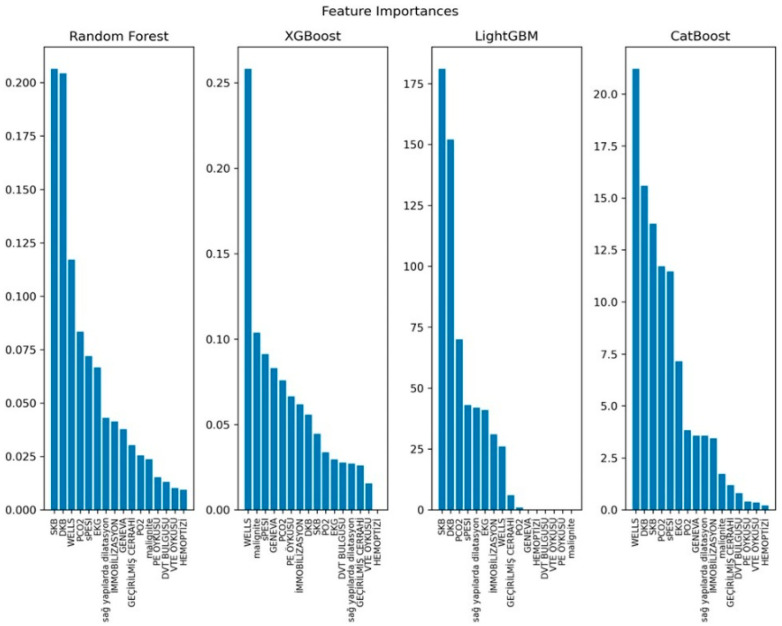
Feature importance plots showing the relative contribution of each clinical variable to mortality prediction across models.

**Figure 7:**
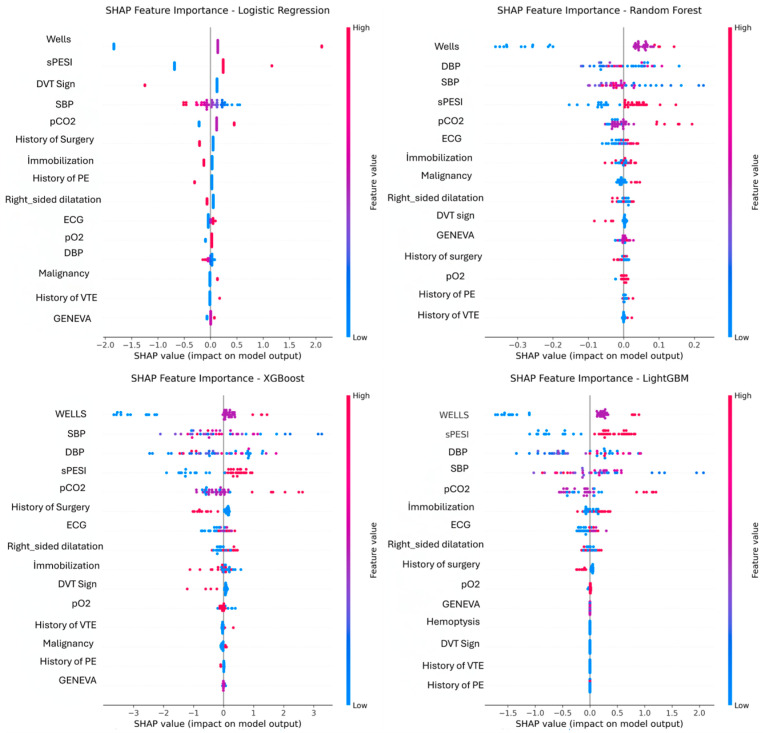
SHAP summary plots of feature contributions to in-hospital mortality prediction in Logistic Regression, Random Forest, XGBoost, and LightGBM.

Feature importance rankings derived from ensemble models are shown in Figure 6, and SHAP summary plots are presented in Figure 7. Systolic and diastolic blood pressure ranked among the top predictors in Random Forest and LightGBM, whereas the Wells score ranked as the leading predictor in XGBoost and CatBoost. Across ensemble models, sPESI and pCO_2_ consistently appeared among higher-importance features, together with ECG abnormalities and right ventricular dilatation.

SHAP summary plots similarly indicated that the Wells score, systolic and diastolic blood pressure, sPESI, and pCO_2_ were among the most influential variables across models. Additional contributions were observed for ECG abnormalities, immobilization, malignancy, pO_2_, and the Revised Geneva score. Overall, feature importance patterns were concordant between model-derived rankings and SHAP-based explanations, supporting consistent interpretability across approaches.

### 3.4. Model Performance Summary

A detailed comparison of accuracy, AUC, precision, recall, F1-score, and specificity is presented in Table 2. Ensemble-based models showed the best overall performance. In the held-out test set, Random Forest and CatBoost achieved the highest accuracy (79.5%) and F1-score (69.0%), with AUC values of 0.880 and 0.870, respectively. XGBoost and LightGBM demonstrated comparable discrimination, with AUCs of 0.880 and accuracies of 77.3%. Logistic Regression achieved an accuracy of 70.5% and an AUC of 0.880, while the k-nearest neighbors model achieved an accuracy of 65.9% and an AUC of 0.850. The support vector machine with a radial basis function kernel showed limited discriminative performance, with an AUC of 0.530 and a recall of 0% on the test set.

### 3.5. Comparison with Clinical Risk Scores

Comparisons versus sPESI were performed in the pre-specified CTPA-confirmed PE subgroup (Table 3), given that sPESI is validated for prognostication in confirmed PE. In the held-out test set, CatBoost achieved the highest discrimination (AUC 0.858), followed by XGBoost (AUC 0.848) and Random Forest (AUC 0.844). In contrast, score-only sPESI and Wells achieved AUC values of 0.721 and 0.618, respectively. DeLong’s test showed that CatBoost, XGBoost, and Random Forest had significantly higher AUC than Wells (all *p* < 0.01). Differences versus sPESI did not reach statistical significance in this subgroup, likely reflecting the limited test set size. The corresponding analysis in the overall suspected-PE cohort is provided as Supplementary Table S1.

Clinical utility and calibration are reported in the Supplementary Material (Supplementary Figures S1 and S2). In the CTPA-confirmed PE subgroup, the ML model showed higher discrimination than score-only sPESI on five-fold out-of-fold predictions (AUC 0.745 vs. 0.632) and lower calibration error (Brier 0.205 vs. 0.228). Decision curve analysis suggested a higher net benefit for the ML model than sPESI and treat-all strategies across clinically relevant threshold ranges.

**Figure 8:**
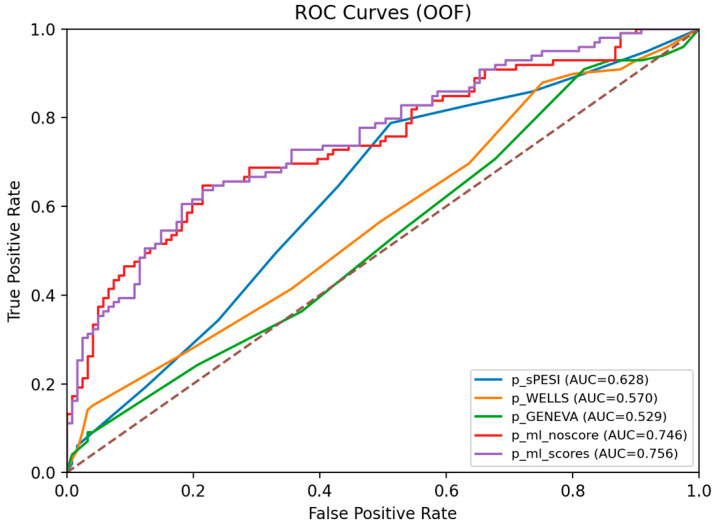
Out-of-fold (5-fold cross-validation) ROC curves for score-only models and the ML framework (± clinical scores).

Out-of-fold predicted probabilities from five-fold cross-validation were used to compare score-only approaches and the ML framework, with and without inclusion of clinical risk-score inputs, as shown in Table 4 and Figure 8. In score-only models, sPESI, Wells, and Revised Geneva achieved ROC-AUC values of 0.628, 0.570, and 0.529, respectively. The ML model without risk-score inputs achieved an ROC-AUC of 0.746, a PR-AUC of 0.744, and a Brier score of 0.207. The ML model, including clinical score inputs, achieved an ROC-AUC of 0.756, a PR-AUC of 0.748, and a Brier score of 0.207. Differences in ROC-AUC relative to the ML model without scores are reported in Table 4.

## 4. Discussion

In this study, the performance of seven machine-learning (ML) algorithms was evaluated for predicting in-hospital mortality among patients with acute pulmonary embolism (PE) using routinely available ED data. Across the evaluated approaches, ensemble-based methods generally exhibited more favorable discriminative performance in this cohort. Taken together, these results support further evaluation in larger, independent cohorts. Accordingly, the findings should be interpreted as hypothesis-generating and require external multicenter validation before clinical use. In this context, these results are broadly consistent with prior studies evaluating ensemble ML models for PE prognostication. Zhou et al. (2025) reported favorable discrimination with Random Forest and emphasized the value of SHAP-based interpretability [28]. Hu et al. (2024) similarly demonstrated strong discrimination with LightGBM in autoimmune disease–associated PE [29]. Ryan et al. (2022) found that XGBoost produced useful discrimination using routinely collected clinical data, while Zhou et al. (2024) reported promising performance in a multicenter federated-learning cohort [15,30]. Overall, prior reports suggest that ensemble learning can perform well across distinct populations and settings, while estimates remain influenced by cohort definitions, endpoints, and feature availability.

Comparable findings have also been reported by El-Bouri et al. (2023), who achieved high discrimination using XGBoost and identified age, malignancy, and hemodynamic instability as major determinants [31]. In this analysis, age, malignancy, oxygenation status (pO_2_), and validated clinical scores (sPESI, Wells, Revised Geneva) similarly emerged as primary predictors of short-term mortality. This pattern is consistent with Yuan et al. (2025), who also identified malignancy and cardiac comorbidities as core prognostic factors [32]. Similar findings have been reported using combined clinical–laboratory predictors and biomarker-derived inputs in boosting-based models [33,34]. Key biomarkers such as D-dimer, troponin, and NT-proBNP were not systematically available in this cohort; biomarker-informed prognostic refinement could not be evaluated. Routinely collected ED data combined with validated clinical scores yielded informative risk discrimination, supporting the feasibility of ML-based prediction tools when advanced laboratory testing is not uniformly available.

Traditional prognostic indices such as PESI and sPESI remain widely used but are constrained by their reliance on a limited set of variables and the exclusion of laboratory and imaging findings [24]. Rather than replacing these indices, embedding established scores within ML pipelines may help synthesize routine physiological signals with familiar clinical constructs. Previous work by Somani et al. (2022), who showed that integrating ECG features into XGBoost enhanced prognostic power, and Su et al. (2022), who improved discrimination using LightGBM combined with hematologic parameters, further supports this conclusion [35,36]. The SHAP analyses in this study corroborate these observations, showing that sPESI, Wells, and Revised Geneva scores are consistently among the most influential predictors, followed by age and oxygenation status. Importantly, model attribution patterns should be interpreted as describing how models used available inputs in this dataset, rather than as causal determinants of mortality. In addition, the overall pattern of results is compatible with the interpretation that routinely collected ED variables can contribute a prognostic signal beyond composite scores, while established scores may provide complementary clinical context.

Beyond single-center studies, multicenter validations and systematic reviews underscore the expanding role of ML in PE risk assessment. Li et al. (2025) described promising results when combining CTPA imaging with electronic health record (EHR) data [37], while Puchades et al. (2024) and Shen et al. (2022) demonstrated that ensemble-based models consistently outperform Logistic Regression and SVM across large datasets [25,38]. The necessity for external validation and methodological standardization is commonly emphasized [23], and emerging real-world applications such as the READ-PE radiology decision support system [39] and transformer-based multimodal deep learning models [40] illustrate ongoing efforts toward clinically oriented implementation while underscoring the importance of transportability, calibration monitoring, and reproducible reporting. Consistent with ED-focused investigations, ML models have generated meaningful predictions using triage-time variables and acute-care features [41,42,43]. In addition, Random Forest models have demonstrated favorable calibration and discrimination compared with Logistic Regression in hospitalized PE cohorts.

Recent reviews reinforce the expanding role of artificial intelligence in the management of acute PE. Henkin et al. (2025) reported that ML algorithms outperform conventional risk scores in high-risk emergency settings, underscoring their potential to support time-sensitive decision-making [44]. Huang et al. (2024) similarly demonstrated favorable calibration and discrimination for Random Forest compared with Logistic Regression in hospitalized patients with PE [43]. Tian et al. (2025) introduced PE-Mind, a prospective deep learning model that predicts PE development in patients with DVT, emphasizing the importance of longitudinal modeling in high-risk populations [45].

Emerging multimodal and EHR-integrated architectures provide additional evidence supporting ML-based PE prognostication. Guo et al. (2024) developed PE-MVCNet, a cross-modal fusion system integrating CT imaging with EMR data, achieving substantially higher AUROC than unimodal models [46]. Naser et al. (2025) similarly confirmed that clinical data–driven ML approaches can be successfully deployed in real-world healthcare environments, reinforcing their translational feasibility [47].

Given the prognostic importance of right ventricular dysfunction in acute pulmonary embolism, it would be valuable to investigate whether multimodal strategies incorporating advanced imaging can more comprehensively characterize the hemodynamic impact of pulmonary embolism across the right ventricle and the cardio-hepatic axis. Evidence from other acute cardiovascular conditions suggests that extracardiac biomarkers derived from cardiovascular magnetic resonance (cardiac MR), such as hepatic T1 mapping, may reflect right-sided loading [48]. Future studies could evaluate whether analogous MR-based phenotyping provides incremental prognostic value in acute PE cohorts.

A related methodological consideration is the distinction between diagnostic and prognostic inference. Because ED clinicians make decisions during the evaluation of suspected PE, model behavior in the suspected-PE pathway is clinically relevant; however, PE-specific prognostic interpretation is most straightforward in confirmed PE. In this context, the comparatively weak and/or conservative behavior observed in some classifiers, particularly SVM with an RBF kernel and, to a lesser extent, KNN, may reflect sensitivity to sample size, class imbalance, feature scaling, and hyperparameter settings. Such characteristics may yield decision boundaries that favor specificity over sensitivity in heterogeneous clinical datasets.

Although ensemble models showed favorable discrimination, threshold-dependent performance indicated a sensitivity–specificity trade-off at the standardized 0.50 threshold; in practice, the operating threshold should be selected to reflect ED risk tolerance and typically prioritize sensitivity in a mortality prediction context.

From a clinical translation perspective, explainability should be viewed as a prerequisite rather than a guarantee of usability. Human-centered evidence indicates that what constitutes a “good” explanation depends on the clinical task, user, and context, and that explanation presentation can meaningfully influence users’ understanding and trust [49,50]. In addition, syntheses focusing on implementability emphasize that workflow alignment, cognitive load, and explanation consistency remain common barriers even when model discrimination is acceptable [51,52]. These observations reinforce that, beyond external validation, prospective human-centered evaluation such as usability testing and workflow integration, is needed before deployment as decision support in emergency care [53].

This study has limitations. It was a single-center retrospective analysis with a modest sample size; therefore, the results represent internal validation only, and transportability requires external validation in independent cohorts, ideally including temporal and geographic validation, before clinical implementation. Excluding patients due to non-retrievable predictors and/or outcome follow-up, most often related to interfacility transfers and fragmented documentation, may have introduced selection bias; because excluded records lacked structured baseline data, an included-versus-excluded comparison was not feasible. In addition, key PE biomarkers such as D-dimer, troponin, and NT-proBNP were not available systematically, which reduces pathophysiologic resolution and limits comparability with biomarker-informed models. Finally, threshold-dependent metrics were reported at a 0.50 probability threshold for standardized reporting; in practice, the operating threshold should be selected to reflect clinical trade-offs, typically prioritizing sensitivity in a mortality prediction setting.

## 5. Conclusions

Future work should prioritize multicenter external validation of ED-based, explainable ML models for mortality risk stratification in suspected acute pulmonary embolism, with prespecified endpoints and standardized reporting of discrimination, calibration, and clinical utility. Prospective implementation studies are needed to assess real-time performance, calibration drift, and the impact on decision curves for disposition, monitoring intensity, and resource utilization.

Model development may be strengthened by incorporating systematically collected biomarkers such as D-dimer, troponin, and NT-proBNP, imaging-derived measures of right ventricular dysfunction, and, when available, longitudinal EHR features. In addition, threshold optimization and cost-sensitive training strategies should be explored to improve sensitivity for high-risk patients while maintaining acceptable specificity in heterogeneous ED populations.

## Figures and Tables

**Table 1:** Demographic, clinical, and laboratory characteristics of patients in the training and test subsets.

Variables	Training Set (*n* = 176)	Test Set (*n* = 44)	*p*-Value
Age, years (mean ± SD)	68.8 ± 15.7	70.3 ± 15.0	0.561
Sex, male, *n* (%)	89 (50.6%)	22 (50.0%)	1.0
Sex, female, *n* (%)	87(49.4%)	22 (50.0%)	1.0
Death, *n* (%)	66 (37.5%)	17 (38.6%)	1.0
Immobilization, *n* (%)	38.0 (21.6%)	14.0 (31.8%)	0.219
Right ventricular dilation, *n* (%)	65.0 (36.9%)	14.0 (31.8%)	0.648
Previous surgery, *n* (%)	38.0 (21.6%)	11.0 (25.0%)	0.777
Malignancy, *n* (%)	18.0 (10.2%)	5.0 (11.4%)	0.787
History of PE, *n* (%)	15.0 (8.5%)	5.0 (11.4%)	0.561
History of VTE, *n* (%)	14.0 (8.0%)	2.0 (4.5%)	0.745
DVT findings, *n* (%)	14.0 (8.0%)	4.0 (9.1%)	0.763
Hemoptysis, *n* (%)	12.0 (6.8%)	2.0 (4.5%)	0.741
sPESI score, mean ± SD	0.7 ± 0.5	0.7 ± 0.6	0.68
Wells score, mean ± SD	1.9 ± 0.5	1.8 ± 0.6	0.227
Geneva score, mean ± SD	1.9 ± 0.4	1.9 ± 0.5	0.532
SBP (mmHg), mean ± SD	119.9 ± 28.8	121.4 ± 25.6	0.446
DBP (mmHg), mean ± SD	71.2 ± 14.8	74.0 ± 18.3	0.686
pO2 (mmHg), mean ± SD	64.9 ± 18.2	66.2 ±17.9	0.606
pCO2 (mmHg), mean ± SD	41.1 ± 14.5	43.2 ± 13.9	0.077
ECG abnormalities, *n* (%)	144.0 (81.8%)	32.0 (72.7%)	0.255

**Table 2:** Predictive performance of machine learning models for in-hospital mortality on the held-out test set.

Model	Accuracy	AUC	Precision	Recall	F1-Score	Specificity
Random Forest	79.50%	0.880	83.30%	58.8%	69.0%	92.60%
CatBoost	79.50%	0.870	83.30%	58.8%	69.0%	92.60%
XGBoost	77.30%	0.880	81.80%	52.9%	64.3%	91.40%
LightGBM	77.30%	0.880	76.90%	58.8%	66.7%	88.90%
Logistic Regression	70.50%	0.880	70.00%	41.20%	51.9%	88.90%
KNN	65.90%	0.850	58.30%	41.25%	48.3%	81.50%
SVM (RBF)	61.40%	0.530	0.00%	0.00%	0.00%	100%

Metrics are reported for the held-out test set. Threshold-dependent metrics were computed using a 0.50 probability threshold for standardized reporting across models; in clinical use, the operating threshold should be selected to reflect the desired sensitivity–specificity trade-off, prioritizing sensitivity in a mortality prediction setting.

**Table 3:** Test-set comparison of ML models and clinical risk scores for predicting in-hospital mortality in the CTPA-confirmed PE subgroup.

Predictor	AUC	Accuracy (%)	F1-Score (%)	*p*-Value (vs. sPESI)	*p*-Value (vs. Wells)
Random Forest	0.844	76.5	75.0	0.090	<0.01
CatBoost	0.858	82.4	80.0	0.075	<0.01
XGBoost	0.848	73.5	74.3	0.129	<0.01
LightGBM	0.789	70.6	70.6	0.460	0.065
Logistic Regression	0.799	79.4	77.4	0.271	0.062
KNN	0.545	55.9	44.4	0.078	0487
SVM(RBF)	0.228	50.0	0.0	0.002	<0.01
sPESI score	0.721	73.5	74.3	Reference	0.300
Wells score	0.618	61.8	38.1	0.300	Reference

Metrics are reported for the held-out test set in the CTPA-confirmed PE subgroup. *p*-values were obtained using DeLong’s test for pairwise comparisons of AUCs versus sPESI and versus Wells. Threshold-dependent metrics were computed using a fixed probability threshold of 0.50.

**Table 4:** Out-of-fold (5-fold cross-validation) performance for clinical risk scores and ML models.

Model	ROC AUC	PR AUC	Brier	ΔROC_AUC
sPESI (score-only)	0.628	0.545	0.227	−0.118
Wells (score-only)	0.570	0.528	0.237	−0.176
Revised Geneva (score-only)	0.529	0.489	0.243	−0.217
ML (no scores)	0.746	0.744	0.207	0.000
ML (+ scores)	0.756	0.748	0.207	0.010

O ROC-AUC, area under the ROC curve; PR-AUC, area under the precision–recall curve; Brier score, calibration error. ΔROC-AUC denotes the difference relative to the ML model without risk-score inputs.

## Data Availability

The data presented in this study is available on request from the corresponding author. The data is not publicly available due to privacy and ethical restrictions.

## Supplementary Materials

The following supporting information can be downloaded at: https://www.mdpi.com/article/10.3390/jcm15041340/s1, Figure S1: Decision curve analysis comparing the ML model and score-only sPESI with treat-all and treat-none strategies in the CTPA-confirmed PE subgroup using five-fold out-of-fold predicted probabilities; Figure S2: Calibration plots for the ML model and score-only sPESI in the CTPA-confirmed PE subgroup using five-fold out-of-fold predicted probabilities; Brier scores are shown in the legend; Table S1: Test-set comparison of ML models and clinical risk scores for predicting in-hospital mortality in the overall suspected-PE cohort (DeLong’s test vs. sPESI and Wells).

## Abbreviations

The following abbreviations are used in this manuscript:

PE	Pulmonary Embolism
ED	Emergency Department
ML	Machine Learning
AUC	Area Under the Curve
SHAP	SHapley Additive exPlanations
sPESI	Simplified Pulmonary Embolism Severity Index
CTPA	Computed Tomography Pulmonary Angiography
ECHO	Echocardiography
ECG	Electrocardiography
VTE	Venous Thromboembolism
DVT	Deep Vein Thrombosis
KNN	K-Nearest Neighbors
SVM	Support Vector Machine
RF	Random Forest
XGBoost	Extreme Gradient Boosting
LightGBM	Light Gradient Boosting Machine
CatBoost	Categorical Boosting
PR	Precision–Recall
