# Supplementary material for: Emergency Department Prediction of In-Hospital Mortality in Suspected Pulmonary Embolism: An Explainable Machine Learning Approach
Source: J Clin Med. 2026 Feb 8;15(4):1340. doi: 10.3390/jcm15041340 (PMC12942220; doi:10.3390/jcm15041340)

ML model performance was compared with established clinical risk scores. On the held-out test set shown in Table 3, sPESI and Wells achieved AUC values of 0.720 and 0.690, respectively. In contrast, ensemble-based machine learning models achieved AUC values ranging from 0.870 to 0.880. Differences between sPESI and the other methods were statistically significant for Random Forest, CatBoost, XGBoost, and LightGBM, with DeLong's test yielding p-values <0.01. Logistic Regression also showed a significant difference compared with sPESI (p <0.05).

Supplementary Table S1. Test-set comparison of ML models and clinical risk scores for predicting in-hospital mortality in the overall suspected-PE cohort (DeLong's test vs sPESI and Wells)

Predictor	AUC	Accuracy (%)	F1-score (%)	p-value (vs sPESI)	p-value (vs Wells)
**Random Forest**	0.880	79.5	69.0	<0.01	<0.01
**CatBoost**	0.870	79.5	69.0	<0.01	<0.01
**XGBoost**	0.880	77.3	64.3	<0.01	<0.01
**LightGBM**	0.880	77.3	66.7	<0.01	<0.01
**Logistic Regression**	0.880	70.5	51.9	<0.05	<0.01
**sPESI score (score-only)**	0.720	65.3	53.0	Reference	—
**Wells score (score-only)**	0.690	62.8	50.0	—	Reference

Footnote: Metrics are reported for the held-out test set within the overall suspected-PE cohort. p-values were obtained using DeLong's test for pairwise AUC comparisons versus sPESI and versus Wells. p-values correspond to AUC comparisons only.

Supplementary Figure S1. Decision curve analysis comparing the ML model and score-only sPESI with treat-all and treat-none strategies in the CTPA-confirmed PE subgroup using five-fold out-of-fold predicted probabilities.

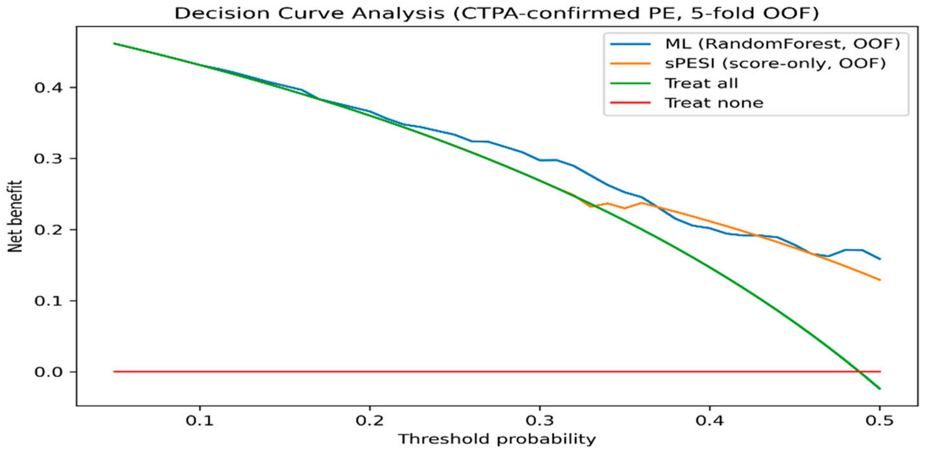

Supplementary Figure S2. Calibration plots for the ML model and score-only sPESI in the CTPA-confirmed PE subgroup using five-fold out-of-fold predicted probabilities; Brier scores are shown in the legend.

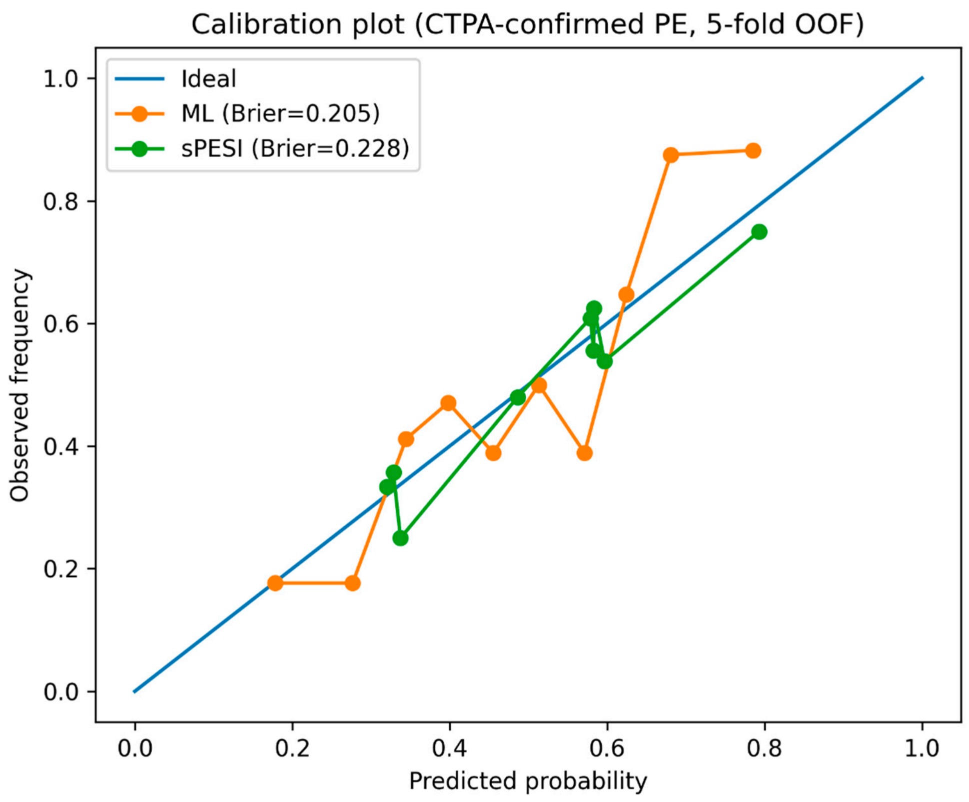

Supplement: Supplementary file 1 [file jcm-15-01340-s001.zip › jcm-4131512-supplementary.pdf]
